# Supplementary material for: Mediodorsal thalamus is required for discrete phases of goal-directed behavior in macaques
Source: eLife. 2018 May 31;7:e37325. doi: 10.7554/eLife.37325 (PMC6010338; doi:10.7554/eLife.37325)
Supplement: Supplementary file 1. — Table shows the amount of food consumed for each animal on each session type. For infusions performed before satiation, mixed effects analysis revealed no significant main effects of food type (F1,15=2.6, p=0.13) or treatment (F0.3,2.1=0.87, p=0.30) nor a food-by-treatment interaction (F1.9,14=0.34, p=0.71). Similarly, for either infusions performed before the probe test, no significant main effects of food type (F1,15=1.15, p=0.30) or treatment (F1.4,10.5=0.005, p=0.99) nor a food-by-treatment interaction (F0.3,2.4=0.07, p=0.52) were detected. Thus, differences in the degree of satiation across session types cannot contribute to the deficits we report. (B) Testing Order for Control Infusions. As described in the methods, to minimize penetrations of the brain, animals received one saline infusion and one sham infusion for each drug. Sham infusions were performed in the same manner as drug infusions except no cannula was inserted. A pair of tests, comprised of one sham infusion and one saline infusion, were performed for each drug (i.e., one pair was performed for MUS, one pair for KYNA). Each pair contained a manipulation performed before satiation and a manipulation performed before the probe session. Furthermore, each pair contained one control with each of the two foods. In the Table, SHAM indicates a sham infusion, SAL indicates a saline infusion. Boxes shaded blue are control infusions for MUS, while those shaded red are control infusions for KYNA. One animal (AN), received an additional control session for KYNA. Data across these two control sessions were summed for analysis. Since LO was not included in the KYNA experiments, this animal only received two control infusions (i.e., those for MUS). (C) Statistical Results for Figure 2. The table shows detailed statistical results for the data presented in Figure 2. Post tests (where appropriate) were Holm-Sidak corrected for multiple comparisons. (D) Tabulated number of objects associated with each food chosen du [file elife-37325-supp1.docx]

**Supplemental File 1**

**Supplemental File 1a. Amount of food consumed (in grams) during selective satiation does not differ across session types.**

|  | Infusion Before Satiation | | Infusion Before Probe | |
| --- | --- | --- | --- | --- |
|  | F1 | F2 | F1 | F2 |
| **Saline Infusions** |  |  |  |  |
| AN | 230 | 151 | 250 | 192 |
| TA | 250 | 30 | 90 | 31 |
| EL | 113 | 174 | 60 | 133 |
| LO | 175 |  |  | 53 |
| x̅ | 192 | 118 | 133 | 102 |
| **MUS Infusions** |  |  |  |  |
| AN | 219 | 124 | 198 | 147 |
| TA | 48 | 33 | 63 | 52 |
| EL | 81 | 133 | 80 | 133 |
| LO | 193 | 77 | 193 | 103 |
| x̅ | 135 | 92 | 134 | 109 |
| **KYNA Infusions** |  |  |  |  |
| AN | 150 | 174 | 250 | 156 |
| TA | 107 | 58 | 161 | 20 |
| EL | 138 | 113 | 36 | 108 |
| x̅ | 132 | 115 | 149 | 95 |

**Supplemental File 1b. Testing Order for Control Infusions.**

|  | Food 1 | | Food 2 | |
| --- | --- | --- | --- | --- |
|  | Before Satiation | Before Probe | Before Satiation | Before Probe |
| AN | SHAM  SAL | SAL | SHAM | SHAM |
| TA | SHAM | SHAM | SAL | SAL |
| EL | SAL | SHAM | SAL | SHAM |
| LO | SHAM | N/A | N/A | SAL |

**Supplemental File 1c. Statistical Results for Figure 2.**

|  | | | | | |
| --- | --- | --- | --- | --- | --- |
|  | Control: x̅ | Treatment: x̅ | Test Statistic | F, t (df), p | Post-Tests* |
| Fig 2C  Object Probe | 0.73 | Before Sat: 0.46  Before Probe: 0.36 | Repeated Measures ANOVA | F = 13.3 (1.1,3.4)  *p =* 0.029 | Control > Before Sat: *p = 0.032*  Control > Before Probe: *p =* 0.035  Before Sat = Before Probe: *p* = 0.13 |
| Fig 2D Consummatory Probe | 0.99 | Before Sat: 0.90  Before Probe: 0.90 | Repeated Measures ANOVA | F = 1.25 (1.3,4.0)  *p* = 0.35 | n/a |
| Fig 2E  Concurrent visual discrimination | 95% | 86.50% | Paired  T-Test | t = 4.09,  df = 3  *p* = 0.026 | n/a |
| Fig 2F  Baseline Preference Ratio | 0.71 | 0.59 | Paired  T-Test  One-Sample T-Test  (Chance = 0.5) | t = 4.28, df=3  *p* = 0.023  Control:  t = 9.81,  df =3, *p =* 0.002  MUS:  t = 3.38,  df =3,  *p =* 0.043 | n/a    n/a |
| Fig 2G  Object Probe | 0.85 | Before Sat: 0.64  Before Probe: 0.42 | Repeated Measures ANOVA | F = 52.3 (1.0,2.1)  *p* = 0.017 | Control > Before Sat: *p = 0.021*  Control > Before Probe: *p =* 0.021  Before Sat = Before Probe: *p* = 0.043 |
| Fig 2H Consummatory Probe | 0.98 | Before Sat: 0.91  Before Probe: 1.0 | Repeated Measures ANOVA | F = 1.11 (1.1,2.1)  *p =* 0.40 | n/a |
| Fig 2I  Concurrent Visual Discrimination | 97.50% | 97.50% | Identical values for all animals under all conditions precludes statistical analysis across treatments | | |
| Fig 2J  Baseline Preference Ratio | 0.68 | 0.7 | Paired  T-Test | t = 0.277, df=2  *p* = 0.81 | n/a |

**Supplemental File 1d. Tabulated number of objects associated with each food chosen during the non-sated probe (F_N_) and during the sated probe (F_D_).**

|  | Infusion  Before Satiation (F1) | | Infusion  Before Satiation (F2) | | Infusion  Before Probe  (F1) | | Infusion  Before Probe (F2) | |
| --- | --- | --- | --- | --- | --- | --- | --- | --- |
|  | **F_N_** | **F_D_** | **F_N_** | **F_D_** | **F_N_** | **F_D_** | **F_N_** | **F_D_** |
| **Saline Infusions** |  |  |  |  |  |  |  |  |
| AN | 14 | 5 | 6 | 4 | 29* | 4* | 8 | 2 |
| TA | 11 | 1 | 8 | 3 | 10 | 2 | 16 | 0 |
| EL | 6 | 1 | 9 | 3 | 12 | 4 | 6 | 0 |
| LO | 19 | 3 | N/A | | N/A | | 2 | 0 |
|  |  |  |  |  |  |  |  |  |
| **MUS Infusions** |  |  |  |  |  |  |  |  |
| AN | 15 | 7 | 8 | 8 | 15 | 9 | 7 | 6 |
| TA | 14 | 4 | 12 | 6 | 14 | 4 | 12 | 6 |
| EL | 12 | 7 | 16 | 8 | 9 | 9 | 11 | 6 |
| LO | 18 | 13 | 2 | 2 | 19 | 9 | 5 | 4 |
|  |  |  |  |  |  |  |  |  |
| **KYNA Infusions** |  |  |  |  |  |  |  |  |
| AN | 10 | 5 | 13 | 5 | 14 | 10 | 6 | 4 |
| TA | 10 | 3 | 11 | 2 | 8 | 5 | 13 | 4 |
| EL | 15 | 4 | 9 | 6 | 15 | 12 | 10 | 3 |
